# Supplementary material for: Stable laser-acceleration of high-flux proton beams with plasma collimation
Source: Nat Commun. 2025 Jan 24;16:1004. doi: 10.1038/s41467-025-56248-4 (PMC11759682; doi:10.1038/s41467-025-56248-4)
Supplement: Supplementary file 1 — Supplementary Information [file 41467_2025_56248_MOESM1_ESM.pdf]

Supplementary Information
Stable laser-acceleration of high-flux proton beams with plasma collimation

M. J. V. Streeter,[1] G. D. Glenn,[2,3] S. DiIorio,[4] F. Treffert,[2,5,6] B. Loughran,[1] H. Ahmed,[7] S. Astbury,[7] M. Borghesi,[1] N. Bourgeois,[7] C. B. Curry,[2,8] S. J. D. Dann,[7] N. P. Dover,[9] T. Dzelzainis,[7] O. C. Ettlinger,[9] M. Gauthier,[2] L. Giuffrida,[10] S. H. Glenzer,[2] R. J. Gray,[11,12] J. S. Green,[7] G. S. Hicks,[9] C. Hyland,[1] V. Istokskaia,[10,13] M. King,[11,12] D. Margarone,[1,10] O. McCusker,[1] P. McKenna,[11,12] Z. Najmudin,[9] C. Parisuaña,[2,14] P. Parsons,[1] C. Spindloe,[7] D. R. Symes,[7] A. G. R. Thomas,[4] N. Xu,[9] and C. A. J. Palmer[1,*]

[1] *School of Mathematics and Physics, Queen's University Belfast, Belfast, BT7 1NN, Belfast UK*
[2] *SLAC National Accelerator Laboratory, 2575 Sand Hill Road, Menlo Park, California, USA*
[3] *Department of Applied Physics, Stanford University, Stanford, California 94305, USA*
[4] *Gérard Mourou Center for Ultrafast Optical Science, University of Michigan, Ann Arbor, MI 48109-2099, USA*
[5] *Institut für Kernphysik, Technische Universität Darmstadt, Karolinenplatz 5, 64289 Darmstadt, Germany*
[6] *Lawrence Livermore National Laboratory, 7000 East Avenue, Livermore, California 94550, USA*
[7] *Central Laser Facility, STFC Rutherford Appleton Laboratory, Didcot OX11 0QX, UK*
[8] *Department of Electrical and Computer Engineering, University of Alberta, Edmonton, AB, T6G1H9, Canada*
[9] *The John Adams Institute for Accelerator Science, Imperial College London, London, SW7 2AZ, UK*
[10] *ELI Beamlines Facility, The Extreme Light Infrastructure ERIC, Za Radnicí 835, 25341 Dolní Břežany, Czech Republic*
[11] *Department of Physics, SUPA, University of Strathclyde, Glasgow G4 0NG, UK*
[12] *The Cockcroft Institute, Sci-Tech Daresbury, Warrington, WA4 4AD, UK*
[13] *Faculty of Nuclear Sciences and Physical Engineering, Czech Technical University in Prague, Prague, Czech Republic*
[14] *Department of Mechanical Engineering, Stanford University, Stanford, California 94305, USA*

This Supplementary Information document provides additional information on the angular variation in proton beam pointing.

Angular variation in proton spectrum

When translating the target parallel to the water sheet surface in the horizontal plane, a horizontal shift in the proton beam axis was observed. We inferred that this behaviour was due to a curvature of the water sheet in the horizontal plane. Supplementary Figure 1a-d shows the spatial profiles for shots with different horizontal offsets (x_T) of the water sheet. At each position, the proton spectrum measured by the TOF spectrometer (Supplementary Figure 1e) remained relatively reproducible, illustrating the capability to steer the proton beam by simply translating the target.

The shift of the beam centroid as a function of x_T followed an overall trend consistent with a radius of curvature of the water sheet of $R_x = 1.3\,$mm (see Supplementary Figure 1f). The reversal of the beam steering direction for $-24 < x_T < 48\,\mu$m indicates the presence of additional curves in the water sheet surface, which lead to a more complex behavior during the lateral position scan [1, 2]. However, at each fixed position the beam pointing was consistent, with a minimum standard deviation of 2.1 mrad at $x_T = -48\,\mu$m and an average standard deviation of 7 mrad over all positions. Translation of the target in the vertical direction by 1 mm did not significantly affect the beam pointing, indicating that the vertical radius of curvature was $R_y \gtrsim 100\,$mm.

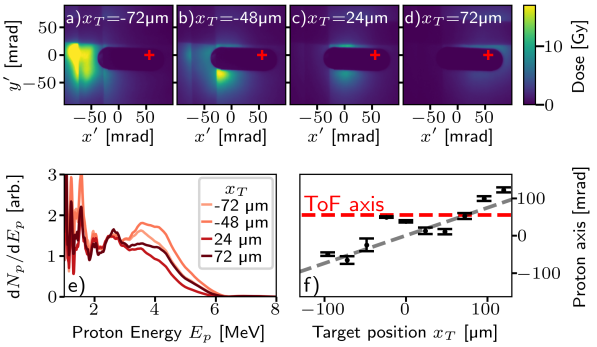

Supplementary Figure 1. **Illustration of beam steering. a-d** Proton dose deposition profiles for different transverse positions of the water sheet (x_T). The ToF spectrometer position is indicated with the red "+", and the average spectra recorded for each case is shown in **e**. **f** The proton beam axis (mean and standard deviation) as a function of target position x_T. The grey dashed line shows a linear fit, which corresponds to a radius of curvature of $R_x = 1.3\,$mm.

* Correspondence email address: c.palmer@qub.ac.uk

[1] F. Treffert, G. D. Glenn, H.-G. J. Chou, C. Crissman, C. B. Curry, D. P. DePonte, F. Fiuza, N. J. Hartley, B. Ofori-Okai, M. Roth, S. H. Glenzer, and M. Gauthier, Physics of Plasmas **29**, 123105 (2022).

[2] C. J. Crissman, M. Mo, Z. Chen, J. Yang, D. A. Huyke, S. H. Glenzer, K. Ledbetter, J. Pedro, F. Nunes, M. L. Ng, H. Wang, X. Shen, X. Wang, and D. P. Deponte, Lab on a Chip **22**, 1365 (2022).
